# Supplementary material for: SeaB is a conserved Salmonella enterica extracellular matrix binding protein involved in biofilm formation and infection
Source: Infect Immun. 2026 Apr 30;94(6):e00754-25. doi: 10.1128/iai.00754-25 (PMC13248724; doi:10.1128/iai.00754-25)
Supplement: Supplemental material — Table S1 and Figs. S1 to S3. [file iai.00754-25-s0001.docx]

Table S1: List of primers

| Primer name | Sequence (5’-3’) | | | | Description |
| --- | --- | --- | --- | --- | --- |
| *seaB*_F | TACGATACCTGGACTTATTACGACAATCCTACCACCGCGCGTGTAGGCTGGAGCTGCTTC | | | For amplification of kanamycin resistance cassette (from pKD4, homologous region in blue) with 40 bases homology to *seaB* | |
| *seaB_*R | ACACCAACGTTTGCAGACCAGTCTTGATCAACATCACCGCGGGAATTAGCCATGGTCCAT | | | For amplification of kanamycin resistance cassette (from pKD4, homologous region in blue) with 40 bases homology to *seaB* | |
| *seaB* check_F | GCACTCTGGCAATCACTTCT | | | Flanking (approx. 200 bases upstream) to *seaB,* to confirm the excision of *seaB* | |
| *seaB* check_R | GAATTCCTGTAGTGGCTTGC | | | Flanking (approx. 200 bases downstream) to *seaB,* to confirm the excision of *seaB* | |
| *seaB* pQE60_F | CGCGCGAGATCTCATATGCACTCCTGGAAAAAGAAAC | | | For amplification of *seaB*, containing a *Nde*I site (Blue), for cloning into pQE60 | |
| *seaB* pQE60_R | GCGCGCGCTAGCAAGCTTACCAGGTATATTTAACACC | | | For amplification of *seaB*, containing an *Hind*III site (Blue), for cloning into pQE60 | |
| T7_F | TAATACGACTCACTATAGGG | | | Forward primer to check pET constructs | |
| T7_R | GCTAGTTATTGCTCAGCGG | | Reverse primer to check pET constructs | | |
| *seaB*_full_notI_F | TTAAGCGGCCGCGACTACTTACGATACCTGGA | Forward primer for cloning the passenger domain and β-barrel domain of *seaB* into pET22b+ with a *Not*I restriction site | | | |
| *seaB*_full_kpnI _R | AACGCCTAGGTTACCAGGTATATTTAACACCAA | Reverse primer for cloning the passenger domain and β-barrel domain of *seaB* into pET22b+ with a *Kpn*I restriction site | | | |

**
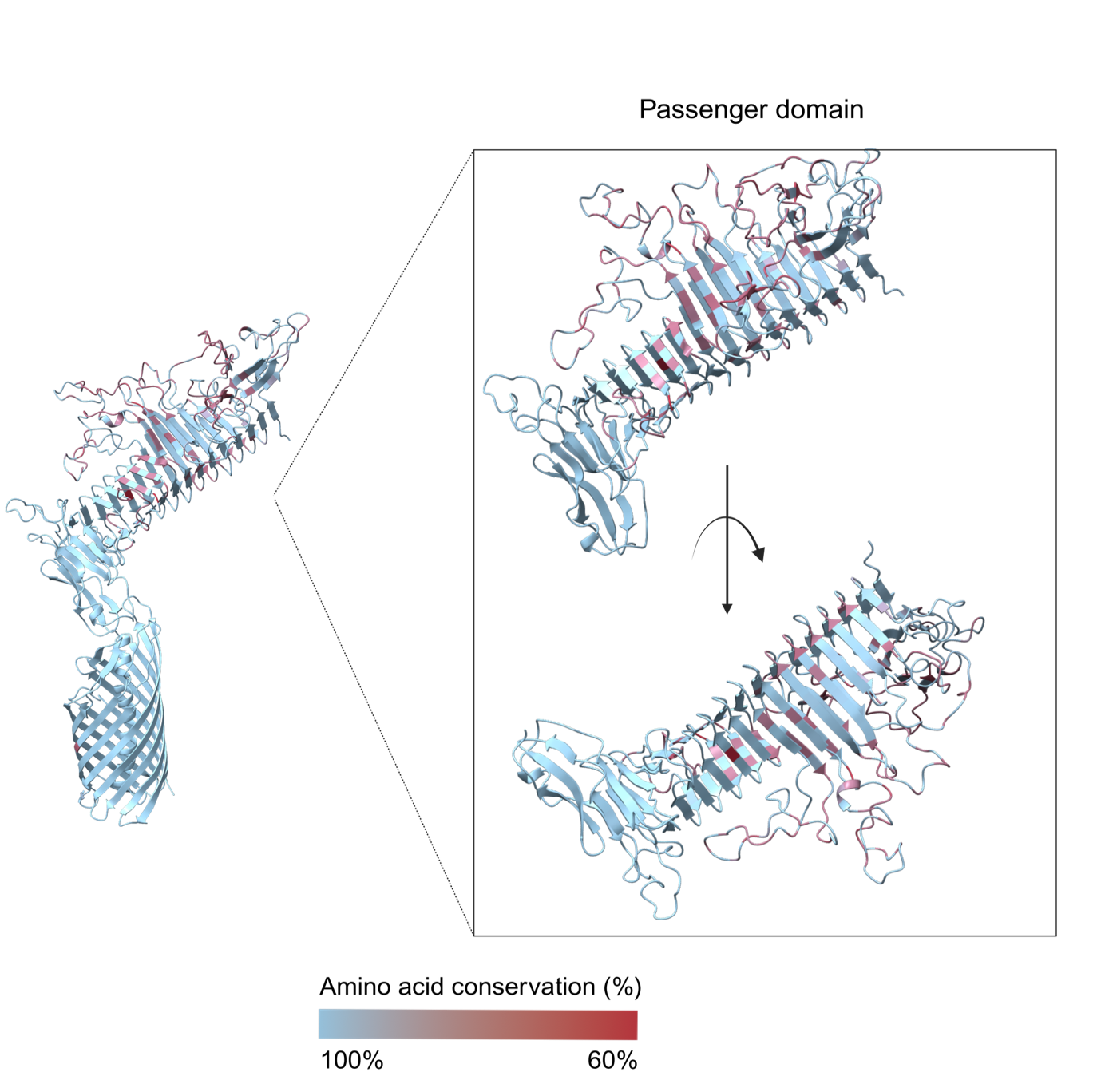
**

**Figure S1. Predicted structure of SeaB homologues.** Alphafold structure of SeaB with highly conserved regions shown in blue. Amino acid conservation for each residue was calculated using exact identity with the R package bio3d (v2.4-5) [61], and percent conservation between 60% to 100% was mapped onto the predicted structure of SeaB using ChimeraX (v1.10), with alignment gaps excluded from visualisation. The predicted domain structure of SeaB has a passenger domain similar to the solved crystal structure of Pertactin with a right-handed β helix, an autochaperone domain, and a 12 stranded C-terminal β-barrel.

**
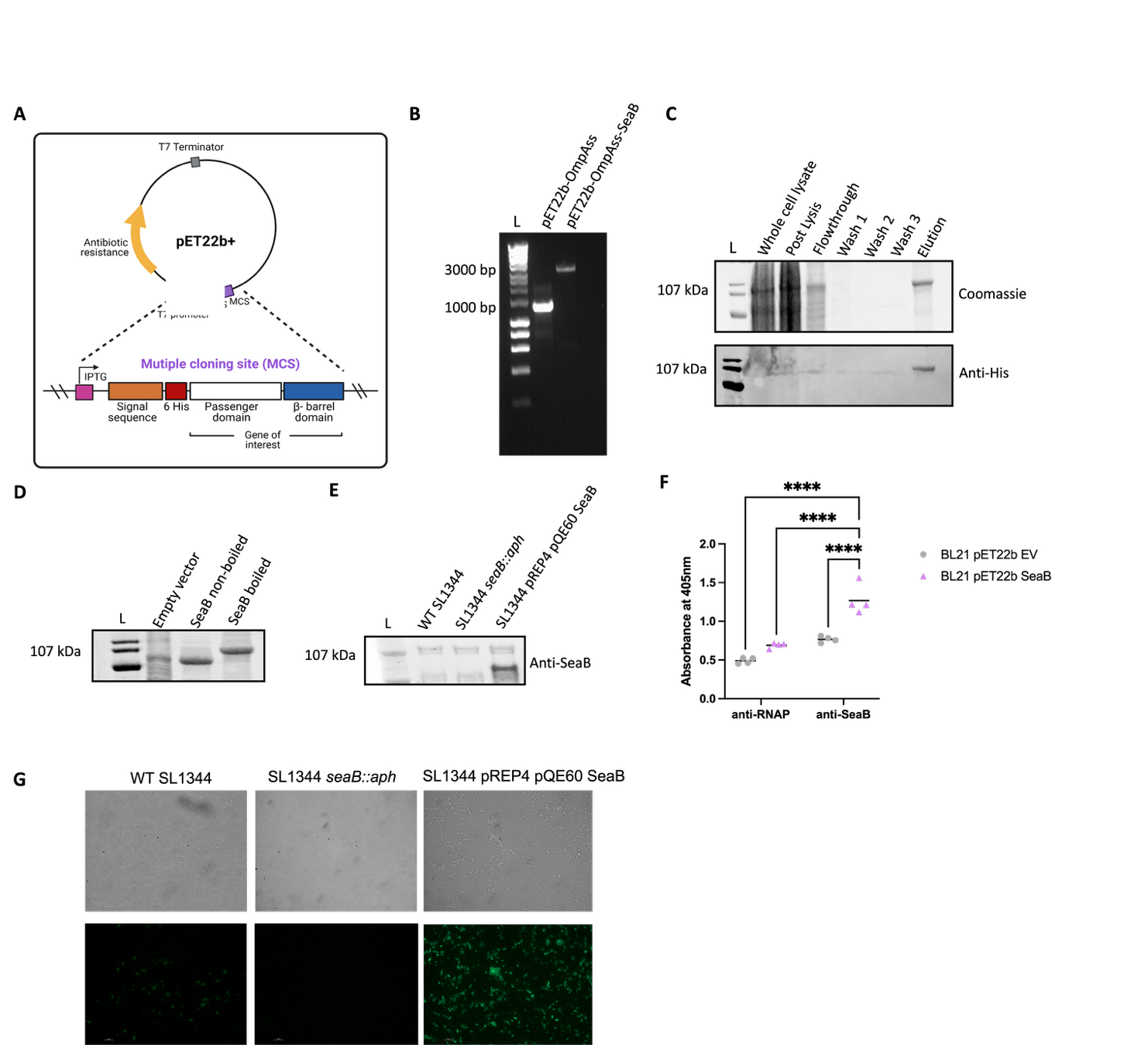
**

**Figure S2. Cloning, purification, and localisation of SeaB.** (A) Overview of the pET22b+ vector used to clone SeaB. OmpA signal sequence was first cloned into the commercially available overexpression vector pET22b+ between the *Nco*I and *Nde*I restriction sites. The passenger domain and β-barrel domain of SeaB was cloned into pET22b+ overexpression vector between *Not*I and *Kpn*I restriction sites in frame with OmpA signal sequence. (B) Gel image of OmpA ss cloned into pET22b+ (left) and SeaB cloned into pET22b+ (right). (C) *E. coli* BL21 P2 encoding SeaB was induced 50 µM IPTG for 16 h at 16˚C. Samples were lysed using the Avestin C3. Following lysis, the protein was incubated with DDM overnight. Samples were purified by Nickel affinity chromatography and analysed by SDS-PAGE (above) and Western immunoblot using anti-His antibodies (below). (D) Purified protein samples (boiled and non-boiled) were run on an SDS-PAGE gel and visualised using Bio-Rad ChemiDoc MP Imaging system. (E) Western immunoblot using anti-SeaB antibodies from outer membrane preparations of *Salmonella* strains (WT, *seaB::aph,* pREP4 pQE60 seaB). (F) Surface localisation of purified SeaB as measured by whole cell ELISA. (G) Localization of SeaB on the cell surface*.* Salmonella strains (WT*,* *seaB::aph*, pREP4 pQE60 seaB) were probed with anti-SeaB primary antibody followed by GAR Alexa Fluor 488. Statistical significance was determined using a two-way ANOVA Sidak’s multiple comparisons test (**** p<0.0001).

**
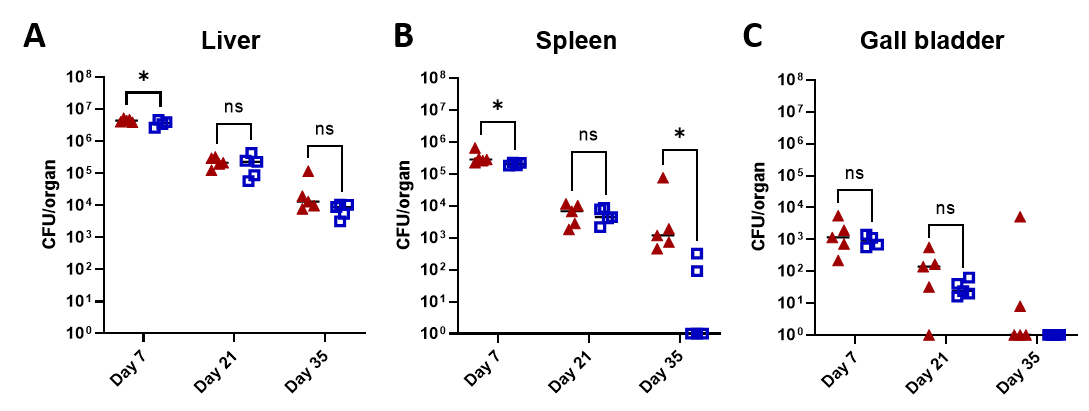
**

**Figure S3. Systemic infection with *Salmonella.*** Bacterial burdens in the (A) liver, (B) spleen and (C) gall bladder of mice infected via the intraperitoneal route for 35 days with WT SL3261 (red closed triangles) and SL3261 *seaB::aph* (blue open boxes). Mice were culled at days 7, 21 and 35 post infection. Statistical significance was determined using Mann-Whitney non-parametric test with correction for multiple comparisons (ns p>0.05, and *p<0.05).
